# Characterization of the complete chloroplast genome of *Platycladus orientalis* (Cupressaceae), an herb to treat lumbar tuberculosis in China

**DOI:** 10.1080/23802359.2019.1699471

**Published:** 2019-12-13

**Authors:** Jun Fei, Shiyuan Shi, Gang Zu, Guihe Han, Zhen Lai, Tianyi Cao

**Affiliations:** Department of Orthopaedics, Zhejiang Integrated Traditional Chinese and Western Medicine Hospital, Hangzhou, China

**Keywords:** *Platycladus orientalis*, Cupressaceae, phylogenetic relationship, chloroplast genome, phylogenetic analysis

## Abstract

*Platycladus orientalis* belongs to the family Cupressaceae that the branches and leaves is an important Traditional Chinese Medicine in China. In this article, the complete chloroplast genome of *P. orientalis* was studied and illustrated to add the more genetic information. The chloroplast genome of *Platycladus orientalis* is 1127,113 bp in length as the circular, which exhibits 120 genes, including 83 protein-coding genes (PCG), 33 transfer RNA genes (tRNAs) and 4 ribosomal RNA genes (rRNAs). The overall nucleotide composition of chloroplast genome is: 32.1% of A, 33.2% of T, 17.9% of C, 16.8% of G and the total AT content of 65.3% and GC of 34.7%. Phylogenetic relationship shown that *Platycladus orientalis* is more closely related to *Thuja standishii* on genetic relationship using the Maximum-Likelihood (ML) method. The chloroplast genome may contribute to the medicinal valuable and evolutionary studies of this species.

*Platycladus orientalis* is also one of the most commonly planted amenity and ornamental conifers, a tradition that goes back many centuries. It is therefore a common tree in parks of towns and cities in much of temperate Asia (Liu 2000). The branches and leaves of *Platycladus orientalis* (Cupressaceae) have been used for thousands of years as Traditional Chinese Medicine, which is mainly distributed in China, Russian, and Korea (Shan et al. 2014). In 2013, *P. orientalis* is classified as 'Near Threatened' in the IUCN Red List of Threatened Species. *Platycladus orientalis* is commonly used in Traditional Chinese Medicine, where it is considered to be one of the 50 fundamental herbs in China. Now, the branches and leaves of *P. orientalis* is mainly used for the treatment of tuberculosis, especially for the upper lumbar tuberculosis. Compared with the traditional incision, the small incision has less trauma and bleeding at the same time of ensuring the operative field of vision. During the treatment, the whole process of side oral treatment is not only anti tuberculosis, reducing the toxic and side effects of tuberculosis drugs, protecting the liver, reducing bleeding, and contributing to postoperative recovery. But, it can treat tuberculosis of upper lumbar vertebrae by modified small incision outside the peritoneum of the 11th rib (Fei and Hu 2017). So, this article had been finished the chloroplast genome of *P. orientalis*, which can be useful for offers the medicinal valuable study in future.

The fresh leaves of *Platycladus orientalis* as the sample was collected from herb market near Zhejiang Chinese Medical University that located at Hangzhou, Zhejiang, China, 30.09 N, 119.89E. The chloroplast genomic DNA of *P. orientalis* was extracted from the fresh root using the modified CTAB method and stored in Zhejiang Chinese Medical University (No. SCMC-ZJU-TCM-04). The chloroplast DNA of *P. orientalis* was purified and sequenced by the sequencer that the collected raw sequences were quality controlled and removed by the FastQC (Andrews 2015). The chloroplast genome of *P. orientalis* was assembled and annotated by the MitoZ (Meng et al. 2019). The chloroplast genome map was generated by the OrganellarGenomeDRAW (Lohse et al. 2013).

The complete chloroplast genome of *Platycladus orientalis* (MN8326262) is a 127,113 base pairs (bp) in length as a circular that don’t have the characteristic quadripartite structure. The chloroplast genome of *P. orientalis* contains 120 genes, which includes 83 protein-coding genes (PCG), 33 transfer RNA genes (tRNAs) and 4 ribosomal RNA genes (rRNAs). The overall nucleotide composition of chloroplast genome is: 32.1% of A, 33.2% of T, 17.9% of C, 16.8% of G and the total AT content of 65.3% and GC of 34.7%.

To analyze the phylogenetic relationship of 14 species chloroplast genomes were used to construct the phylogenetic tree by the Maximum-Likelihood (ML) method. ML analysis of the phylogenetic tree was performed using the MEGA X software (Kumar et al. 2018) with GTR + G + I model and all of the nodes were inferred with strong support by 2000 bootstrap values replicate for each node. The phylogenetic tree was represented using the MEGA and edited using the Evolview online (www.evolgenius.info/evolview) (Subramanian et al. 2019). Phylogenetic relationship shown that *Platycladus orientalis* is more closely related to *Thuja standishii* (KX832627.1) on the genetic relationship (Figure 1). This paper offers the important to the medicinal valuable study and chloroplast genome information of the family Cupressaceae.

**Figure 1.**
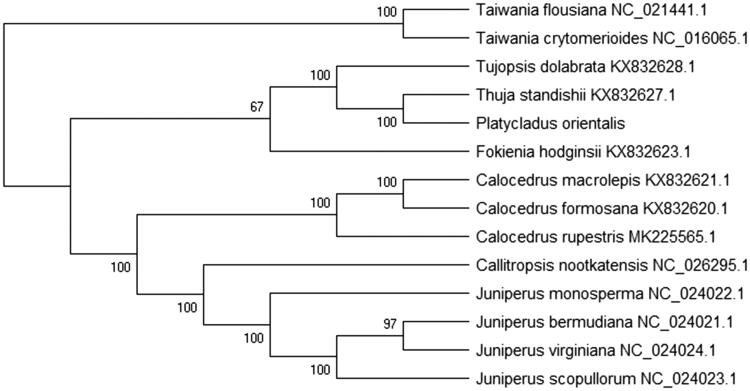
The Maximum likelihood tree of *Platycladus orientalis* based on 15 species chloroplast genomes. Bootstrap support values are shown as numbers on branches. All the nodes are the bootstrap values from 2000 replicates. The NCBI numbers are in the figure.

## References

[CIT0001] Andrews S. 2015. FastQC: a quality control tool for high throughput sequence data. http://www.bioinformatics.babraham.ac.uk/projects/fastqc/.

[CIT0002] Kumar S, Stecher G, Li M, Knyaz C, Tamura K. 2018. MEGA X: molecular evolutionary genetics analysis across computing platforms. Mol Biol Evol. 35(6):1547–1549. 29722887 10.1093/molbev/msy096 PMC5967553

[CIT0003] Liu GW. 2000. Chinese herbal medicine. Peking: Hua Xia Publishing House.

[CIT0004] Lohse M, Drechsel O, Kahlau S, Bock R. 2013. OrganellarGenomeDRAW–a suite of tools for generating physical maps of plastid and mitochondrial genomes and visualizing expression data sets. Nucleic Acids Res. 41(W1):W575–W581. 23609545 10.1093/nar/gkt289 PMC3692101

[CIT0005] Meng GL, Li YY, Yang CT, Liu SL. 2019. MitoZ: a toolkit for animal mitochondrial genome assembly, annotation and visualization. Nucleic Acids Res. 47(11):e63. 30864657 10.1093/nar/gkz173 PMC6582343

[CIT0006] Shan MQ, Shang J, Ding AW. 2014. *Platycladus orientalis* leaves: a systemic review on botany, phytochemistry and pharmacology. Am J Chin Med. 42(03):523–542. 24871649 10.1142/S0192415X14500347

[CIT0007] Subramanian B, Gao S, Lercher MJ, Hu S, Chen W-H. 2019. Evolview v3: a webserver for visualization, annotation, and management of phylogenetic trees. Nucleic Acids Res. 47(W1):W270–W275. 31114888 10.1093/nar/gkz357 PMC6602473

[CIT0008] Fei J, Hu J P. 2017. Surgical treatment of L1-2 tuberculosis by 11th rib extraperitoneal improved mini-incision fusion approach. Chin J Antitubercul. 39(4):370–377.

